# Vancomycin-impregnated decellularized bovine pericardium membranes coating silicone implants: *in-vivo* biocompatibility and antibacterial efficacy in a rat model

**DOI:** 10.1590/acb413326

**Published:** 2026-07-10

**Authors:** Aryzone Mendes de Araujo, Letícia Ramos Dantas, Renato Bespalez, Thais Andrade Costa Casagrande, Paula Hansen Suss, Gustavo Henrique Loesch, Maíra de Mayo Oliveira Nogueira Loesch, Marcelo de Paula Loureiro, Felipe Francisco Tuon

**Affiliations:** 1 Universidade Positivo – Departamento de Biotecnologia – Curitiba (PR) – Brazil.; 2 Pontifícia Universidade Católica do Paraná – School of Medicine – Laboratory of Emerging Infectious Diseases – Curitiba (PR) – Brazil.; 3 Florida Christian University – Orlando (FL) – United States.

**Keywords:** Silicones, Breast Implants, Pericardium, Vancomycin, Infections

## Abstract

**Purpose:**

To evaluate the inflammatory response, fibrotic encapsulation, tissue integration, and antibacterial effectiveness of silicone implants coated with decellularized bovine pericardium membranes, with or without vancomycin impregnation.

**Methods:**

Seventy-two Wistar rats received subcutaneous silicone implants and were allocated to six groups: uncoated silicone under aseptic conditions; uncoated silicone under infected conditions; silicone coated with decellularized bovine pericardium under aseptic conditions; silicone coated with decellularized bovine pericardium under infected conditions; silicone coated with vancomycin-impregnated pericardium under aseptic conditions; and silicone coated with vancomycin-impregnated pericardium under infected conditions. Infected groups were challenged with Staphylococcus aureus and evaluated at day 7. Aseptic groups were analyzed at 30, 60, and 180 days. Histological assessment included semi-quantitative scoring of inflammatory cells, fibroblasts, angiogenesis, implant infiltration, and resorption. Antibacterial activity was quantified by colony-forming unit counts. Data were analyzed using non-parametric statistics.

**Results:**

Vancomycin-impregnated pericardium coatings significantly reduced S. aureus colonization compared with uncoated and pericardium-only implants. At day 7, these coatings markedly attenuated neutrophil infiltration and acute inflammation. At 30 and 60 days, pericardium-coated implants exhibited lower fibroblast scores and enhanced implant infiltration compared with uncoated silicone. At 180 days, vancomycin-impregnated coatings consistently showed reduced fibrotic and inflammatory markers, despite reduced sample size.

**Conclusions:**

Decellularized bovine pericardium coatings modulate the host response to silicone implants, and vancomycin impregnation provides additional antibacterial and anti-inflammatory benefits without impairing tissue integration.

## Introduction

Breast augmentation is one of the most frequently performed aesthetic surgical procedures worldwide and is also widely used in reconstructive surgery following mastectomy^1^. Silicone breast implants are the standard devices employed in these procedures and may be placed using different surgical planes, including subglandular, submuscular, and subfascial approaches^2^. Among these techniques, the subfascial approach has gained increasing acceptance due to superior short- and long-term aesthetic outcomes, faster postoperative recovery, reduced implant displacement caused by pectoral muscle activity, lower postoperative discomfort, and a decreased incidence of intraoperative and postoperative complications^3^. Quantitative evidence supports the advantages of the subfascial approach, demonstrating significantly lower postoperative pain scores—typically ranging from 2 to 4 on the visual analog scale (VAS), compared to 6 to 8 in submuscular procedures—and a faster recovery, with patients returning to daily activities within three to five days. Research indicates a reduction in implant displacement due to the elimination of pectoralis muscle animation, maintaining revision rates for malposition below 2%. Furthermore, the subfascial plane provides a structural ‘internal brassiere’ effect through a fascial layer of approximately 1.5 to 2.5 mm, which effectively masks implant edges and contributes to a lower incidence of capsular contracture (~2–4%) compared to the subglandular technique, while avoiding the trauma and morbidity associated with muscle splitting^4^.

Commercially available silicone breast implants differ in shape (round, conical, or anatomical) and surface characteristics, including smooth, textured, or polyurethane-coated surfaces^5^. Smooth silicone implants, which were widely used in the past, have gradually been replaced due to well-documented disadvantages. One of the major concerns associated with smooth implants is the higher incidence of capsular contracture, a pathological condition characterized by excessive fibrotic capsule formation surrounding the implant, leading to breast hardening, deformity, pain, and, in severe cases, the need for surgical revision^6^. In addition, smooth implants exhibit reduced adherence to surrounding tissues, which may increase the risk of implant displacement or rotation.

To mitigate these complications, alternative surface modifications have been developed, most notably textured and polyurethane-coated implants^7^. Polyurethane-coated silicone implants demonstrate enhanced tissue adherence and a significantly reduced incidence of capsular contracture and seroma formation. Reported rates of capsular contracture with polyurethane-coated implants are markedly lower than those observed with smooth implants, highlighting the importance of surface–tissue interactions in modulating the host foreign body response^8^.

Physiological encapsulation of silicone implants is a normal consequence of wound healing and foreign body response^9^. Following implantation, a thin fibrous capsule forms around the implant, serving as a protective barrier and contributing to implant stabilization^10^. However, in some individuals, this process becomes exaggerated, resulting in pathological capsular contracture. The severity of this response is influenced by multiple factors, including host immune response, genetic predisposition, surgical technique, and implant surface characteristics^11^. Despite advances in implant design, capsular contracture remains the most common late complication associated with breast implants, with reported incidences ranging from below 5% to over 10% in four years of implantation^12^.

Infectious complications, although less frequent than capsular contracture, represent a clinically significant challenge in breast implant surgery^13^. The formation of the fibrous capsule around breast implants is a multifactorial process involving inflammatory, immunological, and mechanical factors. In addition, increasing evidence suggests that bacterial biofilm formation on implant surfaces may contribute to chronic inflammation and the development of capsular contracture. Infection rates following breast implantation range from approximately 1 to 5%, depending on patient-related factors, surgical technique, and perioperative management. The microorganisms most implicated in breast implant–associated infections include *Staphylococcus aureus* and *Staphylococcus epidermidis*, both of which are known for their ability to adhere to biomaterials and form biofilms^14,15^. Gram-negative pathogens such as *Pseudomonas aeruginosa*, as well as *Escherichia coli*, *Enterococcus* spp., *Proteus* spp., and *Klebsiella* spp., have also been reported. Once established, implant-related infections are difficult to eradicate and often necessitate implant removal^16-18^.

Vancomycin is a glycopeptide antibiotic with potent activity against gram-positive bacteria, including methicillin-resistant staphylococci, which are among the most common pathogens associated with implant-related infections^19-22^. While vancomycin is typically administered systemically, local antibiotic delivery strategies incorporated into biomaterials have emerged as a promising approach to reduce infection risk while minimizing systemic exposure. Decellularized bovine pericardium has been increasingly investigated as a biologically derived scaffold due to its favorable biocompatibility, structural integrity, and capacity for cellular integration^23^. When used as a coating material, decellularized pericardium may modulate inflammatory responses, influence collagen organization, and serve as a vehicle for local antibiotic delivery^24^.

While antibiotic-loaded coatings and decellularized biological matrices have been individually explored for implantable biomaterials, the combined use of a decellularized bovine pericardium membrane as both a bioactive interface and a local antibiotic delivery platform applied to silicone implants has not been previously investigated in a longitudinal *in-vivo* model. The primary aim of this study was to evaluate differences in acute inflammatory response and chronic fibrotic encapsulation associated with silicone implants coated with decellularized bovine pericardium membranes in an experimental rat model.

## Methods

This section followed ARRIVE guidelines.

### Decellularized bovine pericardium membranes

Bovine pericardium was collected postmortem from animals destined for human consumption under aseptic conditions. Following harvesting, tissues were immersed in sterile saline solution and maintained at controlled temperatures between 2 and 8°C during transport. In the laboratory, pericardial tissue was carefully dissected to remove adjacent connective tissues, sectioned into standardized strips, and stored under refrigerated conditions until processing.

Decellularization and antibiotic impregnation procedures were performed as previously described^24,25^. The membranes were produced at the Laboratory of Emerging Infectious Diseases, at Pontifícia Universidade Católica do Paraná (PUCPR), following protocols previously approved by the institutional ethics committee and the Brazilian National Research Ethics Commission (CAAE 69676023.7.0000.0020; approval no. 6,495,374). Due to ongoing patent registration, detailed proprietary steps of the decellularization process are not disclosed. After processing, membranes were lyophilized and sterilized using ethylene oxide prior to implantation.

To evaluate the temporal stability of antimicrobial activity, vancomycin-impregnated decellularized bovine pericardium membranes were subjected to an elution assay. Samples were immersed in Milli-Q water and maintained at 37°C for up to seven days, with complete daily replacement of the incubation medium to simulate continuous drug release and depletion conditions. At predefined time points (0 h, 1 h, 6 h, 24 h, 48 h, 72 h, seven days, and 14 days), each membrane was collected and tested for antibacterial activity. The antimicrobial effect was assessed using plating against *S. aureus*, evaluating the presence of bioactive vancomycin released from the pericardial matrix over time. This approach allowed the characterization of the persistence of antimicrobial activity following repeated fluid exchange, simulating a dynamic *in-vivo* environment.

The study was performed at the Laboratory of Emerging Infectious Diseases of PUCPR and Departamento de Biotecnologia (Universidade Positivo).

### Silicone implants

Custom silicone sheets were manufactured by Silimed (Rio de Janeiro, Brazil) specifically for this study, with dimensions of 1 cm × 1 cm and a thickness of 0.1 cm. These dimensions were selected based on previously published experimental models^26-28^. The silicone implants were produced under stringent quality and safety standards consistent with medical-grade manufacturing requirements.

### Ethical Approval

All animal procedures were conducted in accordance with Brazilian Law No. 11,794/2008 and regulations established by the National Council for the Control of Animal Experimentation. The study protocol was submitted to the Institutional Animal Care and Use Committee of Universidade Positivo and approved by it.

### Animals and group allocation

Seventy-two Wistar rats (12 weeks old) were used, comprising 36 males and 36 females, evenly distributed across experimental groups. Animals were allocated into six groups as follows:

1. Silicone implant, aseptic procedure (n = 18; nine males, nine females);
2. Silicone implant coated with decellularized bovine pericardium, aseptic procedure (n = 18; nine males, nine females);
3. Silicone implant coated with vancomycin-impregnated decellularized bovine pericardium, aseptic procedure (n = 18; nine males, nine females);
4. Silicone implant, infected procedure (n = 6; three males, three females);
5. Silicone implant coated with decellularized bovine pericardium, infected procedure (n = 6; three males, three females);
6. Silicone implant coated with vancomycin-impregnated decellularized bovine pericardium, infected procedure (n = 6; three males, three females).

Animals in aseptic groups (1–3) were further subdivided into three postoperative observation periods (30, 90, and 180 days), with six animals per time point (three males and three females). Animals in infected groups (4–6) were euthanized at seven days postoperatively for evaluation of acute inflammatory and infectious responses.

### Surgical procedure

Preoperative analgesia consisted of subcutaneous morphine (0.1 mg/kg). Animals were sedated with inhaled isoflurane and anesthetized via intraperitoneal injection of ketamine (90 mg/kg) combined with xylazine (10 mg/kg). Following dorsal trichotomy and antisepsis with povidone-iodine, sterile surgical fields were established. A longitudinal right scapular–subscapular incision (approximately 3–5 cm) was performed, followed by blunt dissection of the subcutaneous tissue. The corresponding implant was placed according to group allocation.

For infected groups (4–6), contamination was induced by applying 50 µL of a *S. aureus* ATCC 25923 (commonly used for biofilm models) suspension (10^5^ CFU/mL) directly to the implant site prior to closure. Hemostasis was reviewed, and incisions were closed using 5-0 nylon sutures with simple interrupted stitches. Although low-virulence organisms such as *S. epidermidis* and *Cutibacterium acnes* are frequently associated with chronic biofilm formation and capsular contracture, *S. aureus* remains an important pathogen in implant-associated infections and is commonly used in experimental models due to its strong biofilm-forming capacity and reproducibility in infection models.

Animals were housed under standardized conditions with *ad libitum* access to food and water, controlled temperature (20–26°C), and a 12-hour light–dark cycle. Postoperative analgesia included paracetamol (200 mg/mL diluted in drinking water) and subcutaneous tramadol (5 mg/kg every 12 h) for three days. Animals were monitored daily for clinical and behavioral signs of pain or distress, including weight loss (> 10%), altered posture, piloerection, reduced activity, facial grimace, and nesting behavior. Rescue analgesia was administered when predefined criteria were met. Animals showing signs of sepsis were euthanized immediately as a humane endpoint.

Animals were euthanized by isoflurane overdose at predefined time points (7, 30, 90, or 180 days). Implants and surrounding tissues were excised immediately for histological analysis. Biological waste was disposed of via incineration by a licensed third-party service. After implant retrieval, the samples were processed to detach adherent bacteria from the implant surface before CFU quantification, allowing estimation of the viable bacterial burden associated with the biomaterial.

### Histological and morphological analyses

#### Inflammatory response assessment

Excised tissues were fixed in 10% neutral-buffered formalin for 24 hours, processed, and stained with hematoxylin and eosin. Inflammatory response was evaluated based on the presence and intensity of polymorphonuclear and mononuclear infiltrates, edema, vascular congestion, granulation tissue, foreign-body giant cells, and fibrosis, according to the scoring system described by Dantas et al.^23^. Acute and chronic inflammatory components were quantified, and median scores were calculated from five high-power fields per sample.

### Statistical analysis

Continuous variables were expressed as median with interquartile range. Categorical variables were presented as frequencies and percentages. Comparisons among groups were performed using the Kruskal–Wallis’ test for non-normal distributions. All tests were two-tailed, with statistical significance set at *p* < 0.05.

A post hoc power analysis was performed to estimate the achieved statistical power based on the observed effect sizes and sample sizes. Effect sizes were calculated using Cohen’s d for comparisons between groups, based on the difference between means and the pooled standard deviation. The achieved power was then estimated assuming a two-tailed test with a significance level (α) of 0.05. This analysis was applied to histological parameters with measurable variability to assess the sensitivity of the study to detect differences between experimental groups. Analyses were conducted using Statistical Package for the Social Sciences software (version 23.0).

## Results

### Antibacterial effectiveness

**Figure 1:**
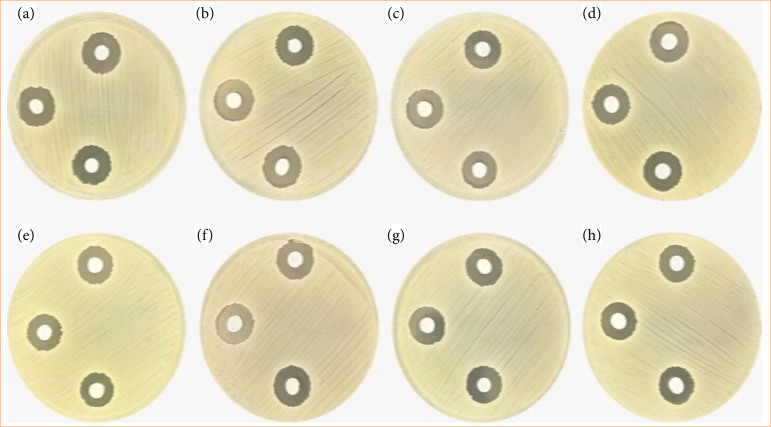
Effects of decellularized pericardium discs loaded with vancomycin on Staphylococcus aureus ATCC®️ 25923 growth assessed by disc diffusion assay. Prior to testing, the discs were immersed in phosphate buffered saline solution for different time intervals: (a) 0 h, (b) 1 h, (c) 6 h, (d) 24 h, (e) 48 h, (f) 72 h, (g) seven days, and (h) 14 days. The inhibition zones indicate the antibacterial activity over time.

Source: Elaborated by the authors.

The elution assay demonstrated sustained antimicrobial activity of the vancomycin-impregnated pericardium over time. At baseline, a strong antibacterial effect was observed, with a mean inhibition zone of 18.33 mm, indicating high initial availability of bioactive vancomycin. This activity remained stable during the early time points, with inhibition zones of 17 mm at 1 and 6 hours, followed by a gradual reduction over time (16.67 mm at 24 h and 15.67 mm at 48 h). Notably, antimicrobial activity persisted despite daily medium replacement, with measurable inhibition zones at 72 hours (16 mm), seven days (14 mm), and 14 days (14 mm). These findings indicated sustained release of vancomycin from the pericardial matrix over time (Fig. 1).

**Figure 2:**
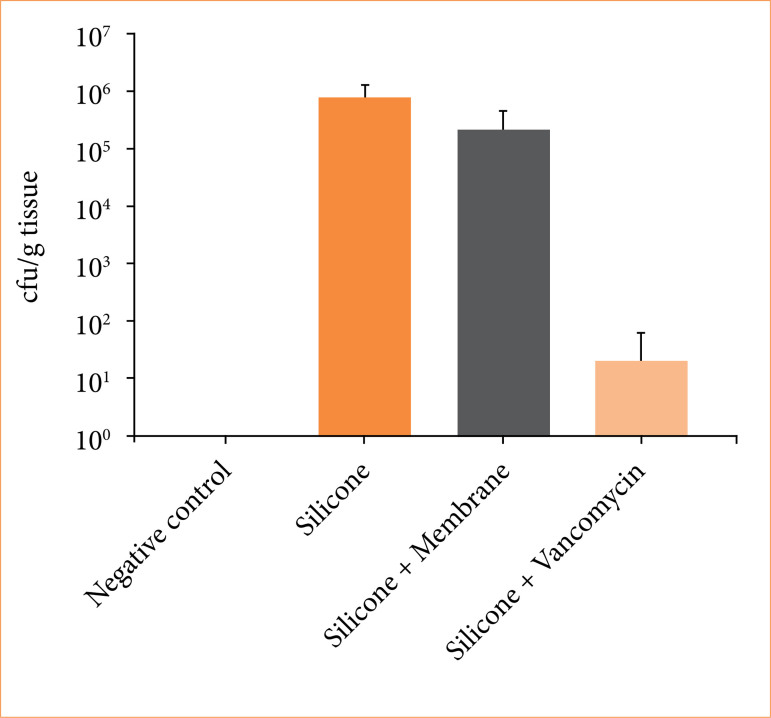
Quantitative analysis of bacterial load (CFU/g of tissue) recovered from infected implantation sites across experimental groups. Uncoated silicone implants and silicone coated with decellularized bovine pericardium demonstrated high levels of bacterial colonization, whereas silicone implants coated with vancomycin-impregnated pericardium showed a marked reduction in bacterial burden, approaching levels observed in the negative control group. Data are presented as median values with interquartile range.

Source: Elaborated by the authors.

Quantitative microbiological analysis revealed substantial differences in bacterial colonization among the experimental groups. Uncoated silicone implants showed high levels of *S. aureus* growth, confirming their susceptibility to bacterial adhesion and proliferation. Similarly, silicone implants coated solely with decellularized bovine pericardium membranes demonstrated elevated bacterial counts, indicating that the biological coating alone was insufficient to prevent microbial colonization. In contrast, silicone implants coated with vancomycin-impregnated decellularized bovine pericardium exhibited a marked reduction in bacterial burden, with CFU values close to those observed in the negative control group. These findings demonstrated that antibiotic impregnation effectively confers antibacterial activity to the pericardial membrane coating, significantly limiting *S. aureus* colonization and supporting its potential role as a preventive strategy against implant-associated infections (Fig. 2).

### Between-group comparisons by time point

**Figure 3:**
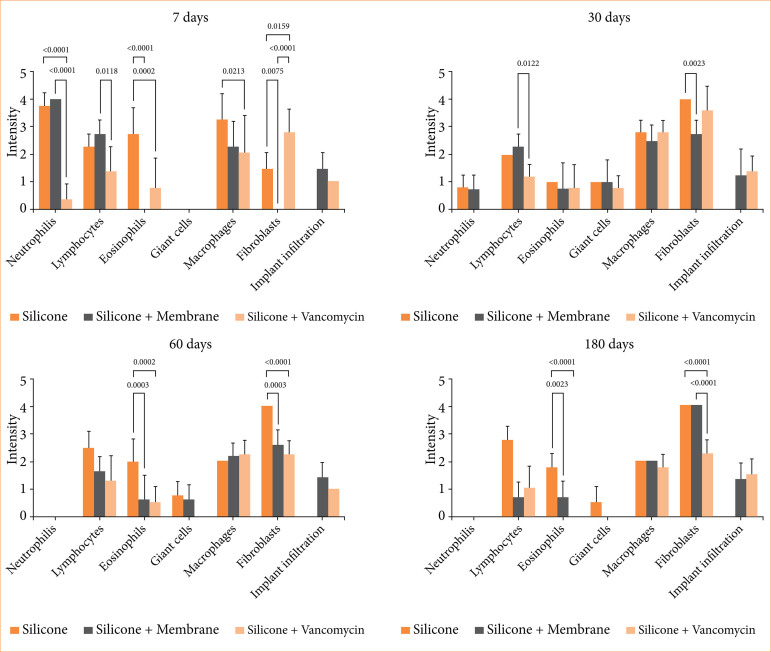
Semi-quantitative analysis of inflammatory and tissue response parameters at day 7, 30, 60, and 180 following implantation. The intensity of neutrophils, lymphocytes, eosinophils, giant cells, macrophages, fibroblasts, and implant infiltration was graded on a standardized scale (0–5). At early time points (seven days), implants coated with vancomycin-impregnated pericardium showed reduced acute inflammatory response compared to control groups, particularly in neutrophil and eosinophil infiltration. Over time, a progressive shift toward tissue remodeling was observed, characterized by increased fibroblast activity and reduced inflammatory cell infiltration. Statistical comparisons between groups are indicated, with significant differences observed across multiple parameters, supporting improved biocompatibility and modulation of the inflammatory response in the vancomycin-treated group.

Source: Elaborated by the authors.

At 7 days, neutrophil scores differed significantly among groups (*p* < 0.05), with vancomycin-impregnated pericardium coatings demonstrating significantly lower neutrophil infiltration compared with both uncoated silicone and pericardium-only coatings (*p* < 0.05 for both). Implant infiltration with macrophages and eosinophils differed between silicone and vancomycin impregnated pericardium at this early stage (*p* < 0.05) (Fig. 3).

At 30 days, significant between-group differences were observed for fibroblast scores and implant infiltration (*p* < 0.05). Uncoated silicone implants exhibited higher fibroblast scores than pericardium-coated implants (*p* < 0.05), whereas vancomycin-impregnated coatings showed greater and more homogeneous infiltration.

At 60 days, fibroblast scores, fibroblasts and eosinophils were higher in silicone alone than silicone protected by pericardium (with or without vancomycin) (*p* < 0.05).

At 180 days, overall group effects were detected for fibroblasts, lymphocytes, eosinophils, infiltration, and reabsorption. However, pairwise comparisons did not consistently reach statistical significance after correction, likely reflecting reduced sample size at late follow-up. Notably, vancomycin-impregnated pericardium coatings consistently showed lower median fibroblast and eosinophil scores.

### Temporal (within-group) comparisons

Within-group temporal analysis demonstrated a significant reduction in neutrophil scores over time in all groups (*p* < 0.05), with the earliest and most pronounced decline observed in the vancomycin-impregnated group. Fibroblast scores increased progressively over time in uncoated silicone implants (*p* < 0.05), whereas pericardium-coated implants exhibited a flatter temporal trajectory, indicating controlled remodeling. Implant infiltration increased over time in coated groups and plateaued at later stages (*p* < 0.05), consistent with stable tissue integration.

A post hoc power analysis performed using the observed effect size for lymphocyte infiltration between the silicone and vancomycin-coated groups (Cohen’s d = 2.58) indicated an achieved statistical power of 87% (α = 0.05), while eosinophil infiltration showed a Cohen’s d of 4.95 (power ≈ 99%), suggesting that the study had sufficient power to detect large differences despite the limited sample size. For other parameters, including neutrophils, macrophages, and fibroblasts, the lack of variability in some groups (standard deviation equal to 0) precluded reliable estimation of statistical power.

## Discussion

This experimental study demonstrated that coating silicone implants with decellularized bovine pericardium membranes significantly modulates the host foreign-body response, and that impregnation of this biological coating with vancomycin provides additional benefits by attenuating early inflammation without compromising tissue integration or long-term biocompatibility. Using a comprehensive histological scoring system across acute, intermediate, and long-term time points, we showed that implant surface modification critically influences the trajectory from initial inflammation to chronic fibrotic encapsulation.

The acute phase following implantation is a critical determinant of long-term implant outcomes^29^. Excessive neutrophil recruitment and early inflammatory amplification have been associated with subsequent fibrotic encapsulation and implant-related complications^30^. In the present study, uncoated silicone implants elicited an intense neutrophil-dominated response at day 7, consistent with a classic foreign-body reaction. Importantly, silicone implants coated with vancomycin-impregnated decellularized pericardium exhibited a marked reduction in neutrophil infiltration, indicating effective attenuation of acute inflammation.

This early effect is likely multifactorial. Local vancomycin delivery may reduce subclinical bacterial contamination, thereby limiting pathogen-associated molecular pattern signaling and downstream inflammatory cascades^31^. In addition, decellularized pericardium provides a biologically derived extracellular matrix scaffold that may reduce nonspecific protein adsorption and macrophage activation compared with inert silicone surfaces^32,33^. Together, these mechanisms likely explain the pronounced reduction in neutrophil scores observed in the antibiotic-impregnated group.

By 30 days, all groups demonstrated a shift from acute to chronic inflammation, characterized by declining neutrophil scores and predominance of lymphocytes and macrophages. This transition reflects normal wound healing and foreign-body adaptation^34^. However, marked differences emerged in fibroblast activity and implant infiltration. Uncoated silicone implants showed higher fibroblast scores, suggesting early fibrotic signaling and capsule formation. In contrast, pericardium-coated implants—particularly those impregnated with vancomycin—exhibited reduced fibroblast scores and enhanced implant infiltration. These effects have been described in *in-vitro* studies, but this is the first description in an *in-vivo* model^35,36^.

These findings suggested that decellularized pericardium alters the balance between inflammation and repair, favoring controlled remodeling rather than excessive fibrotic deposition as previously suggested^23,24,37^. Enhanced infiltration likely reflects improved tissue–implant interaction, which is a hallmark of biocompatible materials and has been associated with reduced rates of capsular contracture in clinical settings^11,38^.

At 60 days, differences in fibrotic behavior became more pronounced. Uncoated silicone implants exhibited persistently elevated fibroblast scores, consistent with progressive fibrotic encapsulation. In contrast, both pericardium-coated groups demonstrated significantly lower fibroblast activity and reduced implant reabsorption scores, indicating attenuation of fibrotic progression. This characteristic is well described because of intense innate response followed by a regulatory response increasing TGF-beta production^26,39^.

Importantly, vancomycin impregnation did not impair tissue integration. Implant infiltration scores remained high in the vancomycin-coated group, demonstrating that local antibiotic delivery did not disrupt host–material interactions. This finding addresses a common concern regarding antibiotic-eluting biomaterials, namely the potential for impaired cellular adhesion or delayed healing^40,41^.

Long-term evaluation at 180 days provides critical insight into the durability of the host response. Although sample size limitations inherent to long-term animal studies reduced statistical power for pairwise comparisons, overall trends consistently favored the vancomycin-impregnated pericardium group. These implants exhibited lower fibroblast scores and reduced indicators of chronic inflammation compared with uncoated silicone implants. Sustained modulation of the fibrotic response suggests that early control of inflammation and microbial burden may have lasting effects on capsule maturation. This observation aligns with emerging evidence that early immune–material interactions can “imprint” long-term foreign-body outcomes, influencing collagen organization, capsule thickness, and mechanical properties.

Implant-associated infection remains a major clinical challenge, particularly in breast and reconstructive surgery. *Staphylococcus aureus* and coagulase-negative staphylococci are frequently implicated in both acute infections and subclinical biofilm formation, which has been linked to capsular contracture. The significant reduction in bacterial burden observed in vancomycin-impregnated pericardium-coated implants supports the concept that local antibiotic delivery can effectively limit early colonization. Beyond infection prevention, reduced bacterial signaling may indirectly attenuate inflammation and fibrosis, providing a mechanistic link between antimicrobial activity and improved biocompatibility. Thus, the benefits of vancomycin impregnation observed in this study likely extend beyond simple antibacterial effects.

These findings are highly relevant to implant-based surgeries, particularly breast augmentation and reconstruction. Capsular contracture and infection remain leading causes of implant failure and revision surgery^1,11,42^. The combination of a biologically derived coating with targeted local antibiotic delivery offers a promising strategy to address both complications simultaneously.

This study has limitations. As an experimental animal model, it may not fully replicate the complexity of human immune responses and mechanical stresses associated with breast implants. Sample size reduction at later time points limited statistical power for some comparisons. Additionally, only a single antibiotic and bacterial species were evaluated. Future studies should assess broader antimicrobial spectra, release kinetics, and mechanical properties of the coated implants. Although fibrosis was assessed using semi-quantitative histological scoring, additional analyses such as capsule thickness measurement, collagen type I/III ratio determination, and polarized light or immunohistochemical evaluation could provide further insight into collagen organization and fibrotic remodeling. These approaches may be considered in future studies to better characterize the fibrotic capsule surrounding the implants. An additional limitation of this experimental model is the absence of physiological mechanical forces that occur in clinical breast implants, including implant movement, tissue loading, and gravitational effects. These biomechanical factors may influence capsule formation and long-term tissue remodeling. However, the controlled environment of the present small-animal model allows the evaluation of early host responses and antibacterial effects under standardized conditions, which are essential for preclinical biomaterial assessment.

A post hoc power analysis suggested that the study had adequate power to detect large differences in key inflammatory parameters, although the limited sample size and low variability in some histological scores prevented reliable estimation for all cellular components.

In summary, this study demonstrated that decellularized bovine pericardium coatings significantly modulate the host response to silicone implants, reducing fibrotic activity and enhancing tissue integration. Vancomycin impregnation further attenuates acute inflammation and provides effective antibacterial protection without compromising biocompatibility. These findings support the use of antibiotic-impregnated biological coatings as a promising strategy to improve the long-term safety and performance of silicone implants and warrant further investigation in translational and clinical studies.

## Data Availability

The data will be available upon request.
